# Injury in aged animals robustly activates quiescent olfactory neural stem cells

**DOI:** 10.3389/fnins.2015.00367

**Published:** 2015-10-08

**Authors:** Jessica H. Brann, Deandrea P. Ellis, Benson S. Ku, Eleonora F. Spinazzi, Stuart Firestein

**Affiliations:** ^1^Department of Biology, Loyola University ChicagoChicago, IL, USA; ^2^Department of Biological Sciences, Columbia UniversityNew York, NY, USA

**Keywords:** stem cell, regeneration, olfactory, renewal, proliferation

## Abstract

While the capacity of the olfactory epithelium (OE) to generate sensory neurons continues into middle age in mice, it is presumed that this regenerative potential is present throughout all developmental stages. However, little experimental evidence exists to support the idea that this regenerative capacity remains in late adulthood, and questions about the functionality of neurons born at these late stages remain unanswered. Here, we extend our previous work in the VNO to investigate basal rates of proliferation in the OE, as well as after olfactory bulbectomy (OBX), a commonly used surgical lesion. In addition, we show that the neural stem cell retains its capacity to generate mature olfactory sensory neurons in aged animals. Finally, we demonstrate that regardless of age, a stem cell in the OE, the horizontal basal cell (HBC), exhibits a morphological switch from a flattened, quiescent phenotype to a pyramidal, proliferative phenotype following chemical lesion in aged animals. These findings provide new insights into determining whether an HBC is active or quiescent based on a structural feature as opposed to a biochemical one. More importantly, it suggests that neural stem cells in aged mice are responsive to the same signals triggering proliferation as those observed in young mice.

## Introduction

The adult nervous system is limited in its capacity to repair itself. Repositories of neural stem cells are found in the subgranular zone, subventricular zone, and in the neuroepithelium lining the nasal cavity. However, the mechanisms responsible for widespread aging-induced diminished neurogenesis are unclear. It is possible the function of the stem cell niche surrounding neural stem cells declines with age, or perhaps the regenerative capacity of the stem cell itself is exhausted. In addition, the fundamental question of whether regeneration in the olfactory system is simply a recapitulation of development or is in fact its own process also remains unanswered (Brann and Firestein, 2014).

The olfactory system is an ideal model system for questions regarding adult neurogenesis, as it is unique in that two of its neuronal populations can be regenerated throughout the life of mammals. One neurogenic population (in the subventricular zone) supplies inhibitory interneurons to the olfactory bulb, while the other (in the nasal neuroepithelium) generates excitatory projection neurons resident in the olfactory epithelium (OE). The basal stem cells that give rise to this latter group in the OE are accessible, easily studied, and are a rich source of adult neural stem cells that may have potential for clinical research (Wetzig et al., 2011). However, in the majority of stem cell populations, the function, potency, or perhaps the remaining replicative cycles, typically declines with increasing age (Signer and Morrison, 2013). It remains an open question if this is also the case for olfactory neural stem cells.

It has been known for over 40 years that the adult olfactory epithelium generates new sensory neurons (Graziadei, 1973). Importantly these sensory neurons, while specialized for transducing chemical stimuli, are true neurons (not specialized epithelial cells) of the Golgi type I, possessing a long axon that projects to targets in the CNS. The olfactory epithelium detects a wide variety of odorants and is a stratified structure consisting of ciliated bipolar olfactory sensory neurons, horizontal basal cells (HBC), globose basal cells (GBC), immature olfactory sensory neurons (OSN_*i*_), mature olfactory sensory neurons (OSN_*m*_), and sustentacular cells (Sus). Both types of basal cells, the HBCs and GBCs, are multipotent and give rise to sensory neurons (Moulton, 1974; Jang et al., 2014). Most OSNs express a single G protein-coupled receptor (GPCR) out of a large family of ~1200 odorant receptors (ORs) (Buck and Axel, 1991; Zhao et al., 1998). OSN axons expressing the same GPCR then travel through foramina in the cribriform plate to synapse in the same loci, called glomeruli, in the main olfactory bulb (OB) (Ressler et al., 1994). As OSNs are generated throughout life, they must therefore continually send new fledgling axons to stereotyped positions in the OB with each cell birth and growth cycle (Graziadei and Monti Graziadei, 1983).

We recently showed that the regenerative capacity of a second olfactory tissue, the vomeronasal organ (VNO), is intact even in very old animals, particularly in response to injury late in life (Brann and Firestein, 2010). In rodents, the VNO primarily detects sexually and socially relevant chemical cues such as conspecific reproductive status and is located inside of a bony cavity and thus is a somewhat protected tissue, as it is not exposed to the environment unless the vascular pump surrounding the VNO is activated (Meredith et al., 1980). In contrast, the OE lines the nasal cavity and is more susceptible to the ravages of environmental insults as it lies directly in the respiratory tract. Like the main OE, the VNO undergoes continuous cell loss and replacement, as has been shown in the hamster, mouse, opossum, rat, and snake (reviewed in Halpern and Martínez-Marcos, 2003). The neuroepithelium of VNO is similar to the OE in that basal cells give rise to sensory neurons and sustentacular cells are integral to maintaining the structure of the VNO. Hence, we were interested in whether these closely related but independent tissues aged differently, as others have found that environmental damage may selectively affect the anterior over that of the posterior OE (Loo et al., 1996).

While the potential of OE basal stem cells to generate sensory neurons has been demonstrated into middle age (Kondo et al., 2010; Suzukawa et al., 2011), and into advanced ages in rats (Loo et al., 1996), until recently, virtually all studies of the regeneration of these sensory neurons have been conducted on young adult animals, where a considerable portion of the ongoing proliferation in the OE appears to be part of an extended program of post-natal growth rather than cell replacement. This was first demonstrated in a landmark study by Weiler and Farbman (1997), where the proliferation of new neurons during the first year of life (in rat) was shown to continue to grow the size of the OE by adding new cells for up to 1 year post-natal, i.e., well into middle age. Thus, it was possible that the documented regenerative capacity of the OE was not so much to meet the challenge of replacing worn out neurons as it was a kind of prolonged post-natal development.

The regenerative capacity of the young OE has been studied quite extensively with several paradigms, including target ablation with unilateral olfactory bulbectomy (OBX), sensory deprivation (via naris occlusion), axotomy, and chemical ablation of the OE. Target ablation and axotomy result in the initial degeneration of sensory neurons in the OE, followed by an upregulation of proliferation of mitotic progenitor cells and subsequent robust regrowth of millions of new sensory neurons in a period of several weeks. The OE is largely reconstituted by approximately thirty days (Costanzo and Graziadei, 1983). In hamsters aged 12–24 months, OSNs send new axons to the OB within 35 days after OBX (Morrison and Costanzo, 1995). Chemical ablation of the OE by compounds such as zinc sulfate, methyl bromide, and methimazole, is also followed by rapid proliferation of basal cells (Matulionis, 1975; Hurtt et al., 1988; Genter et al., 1995, 1996; Williams et al., 2004). An advantage of chemically induced lesions is that the target of regenerating OSNs, the olfactory bulb, remains intact. However, these chemicals may induce anosmia variably if the lesion is incomplete (Xu and Slotnick, 1999; McBride et al., 2003). A chemical lesion with 3,3′-iminodipropionitrile caused a more severe lesion in aged rats than in their younger counterparts (over three times more severe in 21 months of age vs. 10-week-old rats; (Genter and Ali, 1998). Regardless, chemical lesions can be used quite effectively to investigate cellular dynamics in the OE if the extent of the lesion is assessed between age groups.

Recent work has indicated that the OE does change with age. Robinson et al. (2002) demonstrated that the expression of pro-apoptotic genes (procaspase-3 and bax) increased in 24 month old rats when compared to their young 12 week old counterparts. Interestingly, the high levels of both genes in aged rats were similar to that of a post-bulbectomy young animal. However, while proliferation decreases dramatically in rats over the ages of 30–90 days old, the total surface area of the rat MOE increases as the animal ages (Weiler and Farbman, 1997). Therefore, it is unclear if the neurons expressing pro-apoptotic genes discussed above are actually dying; if proliferation decreases and apoptosis increases as a rat ages, one would expect the size of the OE to decrease, which does not occur in rats (Hinds and McNelly, 1981; Weiler and Farbman, 1997). The number of sensory neurons also does not change in guinea pigs (Nakamura et al., 1998) but may occur in mice (Rosli et al., 1999). However, in other species such as dogs and humans, the olfactory epithelium of exhibits sensory neuron loss and sensitivity, perhaps caused by the decrease of enzymes such as cytochrome P-450, critical to detoxification (Getchell et al., 1993; Hirai et al., 1996; Rawson, 2006; Rawson et al., 2012).

Other changes occur with aging in the OE as well. Nestin, a putative neural stem cell marker that also localizes to the endfeet of sustentacular cells (Doyle et al., 2001) as well as to olfactory ensheathing cells (Au and Roskams, 2003), decreases with age. Expression of a different marker of olfactory ensheathing cells as well as the glial cell type in the OE (sustentacular cells), GFAP (Glial Fibrillary Acidic Protein) increases with age (Kim et al., 2005). It is possible that sustentacular cells perform a dual role as both microglia and astroglia, but a marker of microglia, OX-42, did not change with age (Weiler and Farbman, 1998; Kim et al., 2005). The number and appearance of microvillar cells, a somewhat recently discovered cell type in the OE whose function is as of yet unclear, is also altered with aging (Kwon et al., 2005). Additionally, the rate of spontaneous lesions of the olfactory epithelium increases with age, suggesting an age-related dysfunction leading to pathogenesis (Kondo et al., 2009). Aging also diminishes the expression of epidermal growth factor receptor in the OE (Ohta and Ichimura, 2000), and as signaling via this pathway is diminished in the subventricular zone (Enwere et al., 2004), it is possible a similar decrement in function is also occurring in the OE.

Overall, proliferation declines with age, and in middle ages continues at low levels presumably to replace the occasional dying neuron. Beyond this observation there is a paucity of experimental evidence regarding regeneration in older animals and questions about the functionality of newborn neurons in aged animals remain unanswered. Here, we endeavored to extend our results from the VNO into the OE by investigating the rate of neurogenesis in aged mice and if newborn neurons born in aged mice mature properly. Finally, we examine the ability of an apparently quiescent stem cell population to be reactivated in aged mice to repopulate the olfactory epithelium following injury. In the course of our study we also observed a novel transition in the morphology of HBCs as they switch from a quiescent to a proliferative phenotype. Hence, we demonstrate that the regenerative capacity of the OE is extremely robust, even at very advanced ages.

## Materials and methods

### Animal care and sources

C57BL/6 male mice, aged 1–24 months, were obtained from the NIA Aged Rodent Colony. All experimental procedures were in compliance with NIH guidelines and were approved by the Columbia University Institutional Animal Care and Use Committee.

### Antibodies

Antibodies used for immunohistochemistry were those recognizing BrdU (5-bromo-2′-deoxyuridine; mouse monoclonal; GE Healthcare Biosciences, Piscataway, NJ; catalog number RPN202; clone BU-1; dilution 1:30), cytokeratin 5 (Krt5; rabbit monoclonal; Epitomics/AbCam, Burlingame, CA; catalog number 2290-1; dilution 1:200), olfactory marker protein (OMP; goat polyclonal; Wako, Richmond, VA; catalog number 544-10001-WAKO; dilution 1:1500) and PCNA (proliferating cell nuclear antigen; mouse monoclonal (IgG2a kappa); Abcam, Cambridge, MA; catalog number Ab29; dilution 1:200).

### Immunohistochemistry (IHC)

Mice were anesthetized by intraperitoneal injection with a mixture of ketamine/xylazine (100 mg/kg and 8 mg/kg, respectively) and perfused transcardially with heparinized (4 unit/mL) phosphate buffer saline (PBS) (pH 7.4) followed by 4% paraformaldehyde (Sigma, St. Louis, MO) in 0.1 M phosphate buffer (PB; pH 7.4). Olfactory epithelia were dissected, decalcified, cryoprotected (30% sucrose) and frozen in OCT. Cryosections (12 μm) were incubated in blocking solution containing 0.5% Triton X-100 and 5% normal donkey serum in 0.1 M PB for 1 h at room temperature. Antigen retrieval was performed with Retrievagen A (pH 6.0, catalog number 550524, BD Biosciences, San Jose, CA) or Retrievagen B pH 9.5, 550527; BD Biosciences) if necessary. Primary antibody incubation was performed overnight at 4°C. Sections were then incubated with Alexa Fluor 488 and 594 conjugated secondary antibodies (Molecular Probes, Eugene, OR; 1:750) for 2 h at room temperature. TOTO-3 (Invitrogen, Carlsbad, CA; 1:10,000) was added in a wash step after secondary incubation for visualization of nuclei. Sections were mounted in Vectashield (Vector Laboratories, Burlingame, CA) to prevent photobleaching. Optical sections (1 μm) were taken through the depth of the section with a Zeiss LSM700 (Thornwood, NY) confocal microscope and analyzed with ImageJ software. Figures were assembled with Adobe Photoshop and Illustrator software.

### Lesions of the olfactory epithelium

To chemically ablate the olfactory epithelium, methimazole (Sigma, St. Louis, MO) was administered via single intraperitoneal injection (50 mg/kg). To surgically ablate the OE by severing the axons of mature sensory neurons, OBX was performed according to standard methods (Brann and Firestein, 2010). Briefly, mice were anesthetized by intraperitoneal injection with a mixture of ketamine/xylazine (100 mg/kg and 8 mg/kg, respectively). A small hole was cut in the frontal bone over the right olfactory bulb with a dental drill, and the bulb was removed by aspiration. The cavity was filled with Gelfoam (Pfizer Pharmacia and Upjohn Co., Kalamazoo, MI) to control bleeding, and the skin over the wound was sutured closed with Vetbond (3M, St. Paul, MN).

### Proliferation assay

Proliferation was assessed by BrdU (Sigma, St. Louis, MO) injection and was detected with an antibody recognizing BrdU (described above). For acute labeling experiments, 100 mg/kg BrdU was injected 2 h prior to sacrifice. For maturation studies (see below), two injections 2 h apart of 50 mg/kg BrdU were given; the animals were then sacrificed 30 days following injection. For OBX studies, 100 mg/kg was injected 2 h prior to death on the fifth or thirtieth day after OBX was performed.

### Maturation assay

Cryosections were prepared as described above (see *Immunohistochemistry*). In all animals, at least 10 coronal sections throughout the OE were examined to yield a minimum of 25 BrdU positive cells examined per animal. Optical sections (1 μm) were taken with a 40x objective through the depth of the section with a Zeiss LSM700 confocal microscope and analyzed with ImageJ software. Complete overlap in all visual planes with a nuclear marker (TOTO-3) was required in order to verify that examination was of a single BrdU-labeled cell. Z-projections through the middle of BrdU/TOTO-3 positive nuclei were then made to verify that OMP labeling surrounded the entire nucleus. Cells that exhibited partial labeling with OMP or those in which the plane of cryosection bisected the nucleus were not included in the analysis.

### Stereology and quantification

Thin cryosections (12 μm) were prepared as described above (see *Immunohistochemistry*). In all animals, every fifth section (60 μm increments) of olfactory epithelia were processed for BrdU and OMP immunohistochemistry, counted immediately with a Leica DMR microscope (Leica Microsystems, Bannockburn, IL) to minimize effects of photobleaching, and photographed with a SPOT digital camera (Diagnostic Instruments, Sterling Heights, MI). The area of OMP immunoreactivity was used to define the reference space and was measured with ImageJ (NIH) software to allow for normalization of counts. Counts were performed by a trained experimenter blind to condition in an unbiased, random manner with systematic sampling. The total number of BrdU-positive cells was not calculated but rather the number of BrdU-positive cells per unit area, as BrdU labeling observed at the dosages utilized here is a rare event. For quantification of HBC number, at least two positions along the dorsal septum in each animal were imaged with a Zeiss LSM 700 confocal microscope. The number of Krt5+ cells was counted with NIH ImageJ Cell Counter, the length of the counted OE was recorded, and the number of cells per mm was calculated for each animal. Three to five animals were analyzed per group.

### Statistical analysis

All statistical analyses were performed with GraphPad Prism software (GraphPad Software, La Jolla, CA). For analysis of basal proliferation levels, all data were analyzed for statistical significance by One-Way ANOVA and Student Newman-Keuls (SNK) pairwise multiple comparison between age groups. For maturation studies, data were expressed as a ratio defined by the number of cells that were both OMP-positive and BrdU-positive divided by the number of BrdU-positive cells, regardless of OMP status. Ratio data were analyzed with a One-Way ANOVA and SNK multiple comparison between age groups. For OBX studies, olfactory epithelium measurements were performed in the same tissue section to control for effects of position, permitting the analysis of control epithelium (olfactory bulb intact) and contralateral lesioned epithelium (olfactory bulb removed) in a single section. Data were analyzed with Two-Way repeated measures ANOVA for the effects of age and treatment (OBX), followed by Bonferroni multiple comparisons test between lesion and non-lesion control for each age group. For HBC cell number and morphology studies, data were analyzed by Two-Way ANOVA for the effects of age and treatment (methimazole lesion).

## Results

### Proliferation declines with age in normal intact olfactory epithelium

We first examined whether proliferation in the adult olfactory epithelium continues to decline with age. Although previous work has shown that proliferation does decline, these studies examined animals only to middle ages. Kondo et al. recently found that the rates of proliferation and cell death decline in animals up to 16 months of age (Kondo et al., 2010) but we sought to extend these findings to mice up to 24 months of age given that the expression of odorant receptors may vary significantly throughout the entire lifespan of mice (Rodriguez-Gil et al., 2010; Khan et al., 2013) and the C57BL/6 mouse lifespan ranges up to an extreme of ~30 months (Konen et al., 1973).

**Figure 1:**
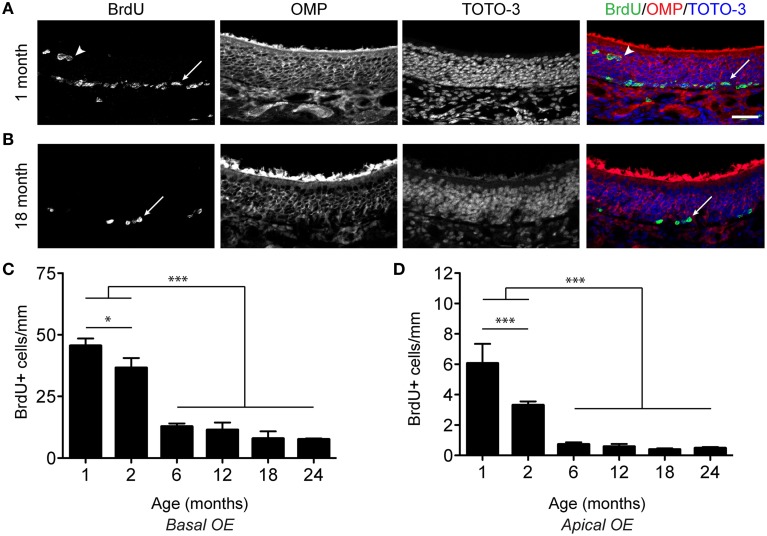
**Proliferation decreases in the olfactory epithelium with age. (A,B)** Incorporation of BrdU in the OE of a 1 month **(A)** and 18 month **(B)** old male mouse. 100 mg/kg BrdU was administered via intraperitoneal injection 2 h before perfusion. Mature neurons are identified by their expression of OMP (red in merged image), while all nuclei are labeled by TOTO-3 (blue in merged image). Cells at the base of the epithelium (arrows) as well as cells in the apical surface of the OE (arrowheads), likely resident in the sustentacular cell layer, incorporate BrdU. Scale bar, 50 μm. **(C)**, Counts of BrdU-positive cells (per mm) were made for basal OE. BrdU-positive cells per mm are represented as the mean ± SEM across age groups ranging from 1 month to 24 months of age. **(D)**, As in **(C)** but for the apical OE. All data were analyzed for statistical significance by One-Way ANOVA and SNK pairwise multiple comparison between age groups, ^*^p < 0.05; ^***^p < 0.0001. *N* = 5–8 animals per group. Ages tested included 1, 2, 6, 12, 18, 24 months of age.

The rodent olfactory epithelium covers the surface of several bony turbinates near the nasal septum. For quantification purposes, the epithelium was divided into zones based upon anatomical landmarks and anterior/posterior position. We used an acute 5-bromo-2′-deoxyuridine (BrdU) labeling paradigm to label proliferative cells, where mice were injected with 100 mg/kg BrdU 2 h before sacrifice. As we observed BrdU incorporation in both basal (Figures 1A,B; arrows) and apical (Figures 1A,B; arrowheads) regions of the epithelium, we quantified these populations separately. Proliferation along the basal extent is primarily associated with the generation of neurons, while that in the apical extent is likely associated with the generation of sustentacular cells. We found that proliferation declines precipitously with age regardless of zone and hence pooled the data depicted in Figure 1. This effect was observed in both the basal and apical regions of the epithelium (Figures 1C,D).

### Proliferation increases in aged olfactory epithelium following lesion

While we observed an inverse relationship between age and the level of proliferation in an intact epithelium, it was not clear from these data whether the proliferative capacity of neural stem cells also declined in aged animals. We therefore asked whether a lesion challenge, namely unilateral OBX, would result in an increase in proliferation in the OE. Lesion of the axons extending from the olfactory epithelium to the olfactory bulb by OBX results in a wave of apoptotic cell death that selectively targets all mature neurons in the epithelium within 5 days of the lesion. In young animals the robust regenerative capacity of the basal cells repopulates the epithelium with > 8–10 million new neurons within 3–4 weeks (Costanzo and Graziadei, 1983; Schwartz Levey et al., 1991; Caggiano et al., 1994; Kastner et al., 2000; Yoshida-Matsuoka et al., 2000; Carter et al., 2004).

**Figure 2:**
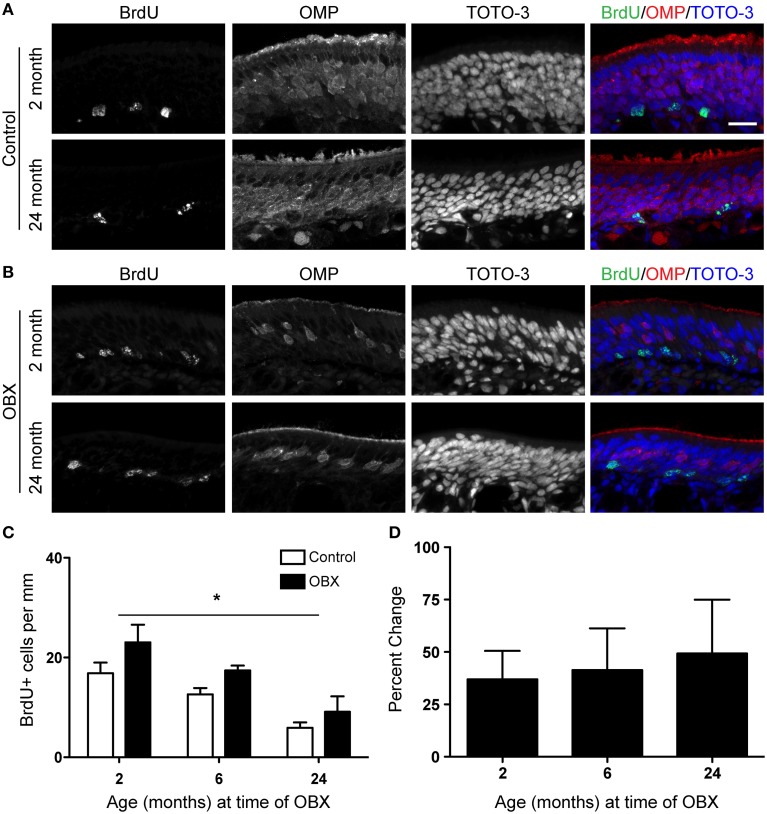
**Acute unilateral OBX lesion induces proliferation in the olfactory epithelium in young and aged mice**. BrdU was administered 2 h before sacrifice on day 5 following OBX. **(A)** BrdU (green), OMP (red), and TOTO-3 (nuclei; blue) in non-lesioned control 2 month old (top) and 18 month old (bottom) olfactory epithelium. **(B)** As in A but for 2 month old (top) and 18 month old (bottom) olfactory epithelium 5 days following OBX. Scale bar, 20 μm. **(C)** Proliferation declined with age in both lesion and non-lesion OE. However, quantification of BrdU incorporation reveals a significant increase (^*^p < 0.05) in proliferation in the OE following acute OBX (black bars) over that in the non-lesion control (white bars; *n* = 3–5 animals per group) in all age groups. All data were analyzed by Two-Way repeated-measures ANOVA for the effects of surgery (*p* < 0.05) and age (*p* < 0.05). **(D)** The percent change between lesion and non-lesion of the number of cells incorporating BrdU, analyzed by One-Way ANOVA, was not different between the age groups tested (*p* > 0.05).

We first performed unilateral OBX in mice aged 2, 6, and 24 months. Five days after surgery, mice were injected with 100 mg/kg BrdU 2 h before sacrifice and their olfactory epithelia were examined for BrdU incorporation (Figures 2A,B). Following stereological quantification, although proliferation did decline with age, we observed proliferation significantly increased throughout the epithelium in all age groups after OBX as compared to non-lesioned controls (Figures 2C,D). When we examined the percent change between lesion and non-lesion for each age, an index of the proliferative capacity of this tissue, we observed no change with age (Figure 2D). These data demonstrate that, although proliferation required for normal neuronal turnover slows with age, the capacity for robust proliferation remains when challenged by lesion.

**Figure 3:**
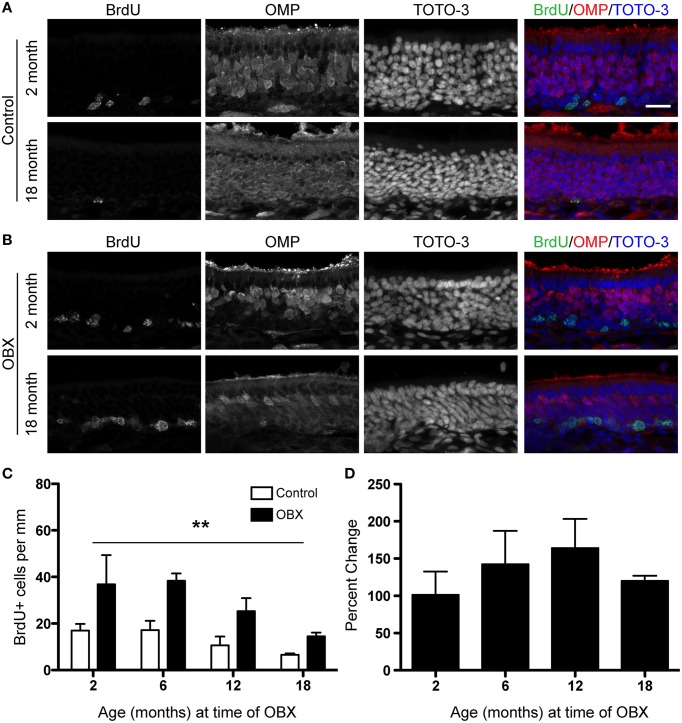
**Sustained unilateral OBX lesion induces increased proliferation in the OE in young and aged mice**. BrdU was administered 2 h before sacrifice on day 30 following OBX. **(A)** BrdU (green), OMP (red), and TOTO-3 (nuclei; blue) in non-lesioned control 2 month old (*top*) and 18 month old (*bottom*) olfactory epithelium. **(B)** As in **(A)** but for 2 month old (top) and 18 month old (bottom) olfactory epithelium 30 days following OBX. Scale bar, 20 μm. **(C)** Quantification of BrdU incorporation reveals a significant increase (^**^p < 0.0001) in proliferation in the OE following sustained OBX (black bars) over that in the non-lesioned control (white bars) regardless of age (*n* = 3–4 per group). All data were analyzed by Two-Way repeated-measures ANOVA for the effects of surgery (*p* < 0.001) and age (*p* > 0.05). **(D)** Percent change between lesion and non-lesion of the number of cells incorporating BrdU, analyzed by One-Way ANOVA (*p* > 0.05) reveals no effect of age on the percent change response.

The OBX lesion model requires the removal of the olfactory bulb and thus the target for the re-growing neurons is absent. In younger animals, this sustained lesion results in the continuous turnover of sensory neurons as newly generated cells fail to find a target and die. We were therefore interested in these continuing effects in the aged epithelium. To test this, we performed unilateral OBX in mice aged 2, 6, 12, and 18 months. We then waited 30 days after surgery before injecting mice with 100 mg/kg BrdU 2 h before sacrifice. We also performed surgeries in 24 month old animals, but were not able to include this group because an insufficient number for statistical analysis survived 30 days following OBX. We did observe that in all age groups tested, proliferation was increased throughout the lesioned epithelium (Figures 3B,C) over that of non-lesioned controls (Figures 3A,C) as was observed with the acute lesion (Figure 2). The percent change between lesion and non-lesion tissue was not measurably different between the age groups tested (Figure 3D). These data together demonstrate that the capacity for long-term, continuous, proliferation from adult stem cells in the olfactory epithelium is undiminished even in very old animals.

### Fate of newborn cells is not altered with aging

**Figure 4:**
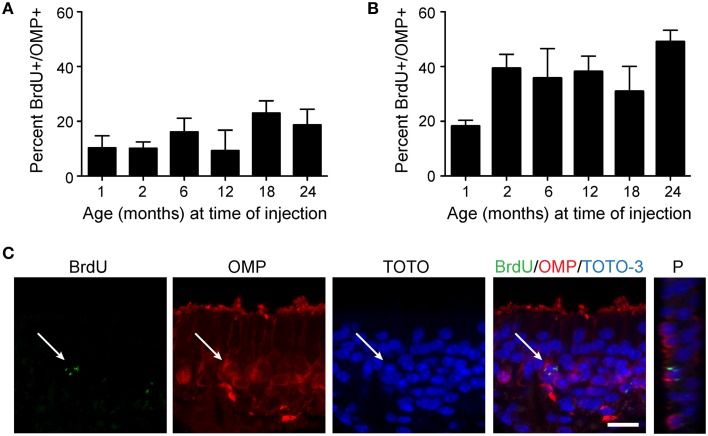
**Newborn neurons become mature olfactory sensory neurons at the same rate in young adult and aged mice**. Mice were injected twice with 50 mg/kg BrdU 2 h apart, tissue was harvested either 10 days **(A)** or 30 days **(B)** following injection, and cells incorporating BrdU were analyzed for the co-expression of OMP, an indicator of olfactory sensory neuron maturity. The percentage of cells incorporating BrdU that reach maturity (OMP-positive) is consistent across all age groups examined (1, 2, 6, 12, and 24 months of age; One-Way ANOVA, *n* = 4–5 animals per group, *p* > 0.05) at both 10 days and 30 days following administration of BrdU. **(C)** Representative confocal images of a BrdU-positive cell (green) surrounded by OMP (red) and Z-projection (P) of BrdU-immunoreactive cell. Scale bar, 20 μm.

Since we observed that proliferation does decline with age, but also that the olfactory epithelium is quite capable of responding robustly to a lesion, we next asked whether the progeny of neural stem cells in aged animals were capable of maturing at the same rate as that observed in young animals. BrdU was again used to label proliferative cells, but to avoid possible cellular toxicity, two injections of a lower dose of BrdU (50 mg/kg) were administered 2 h apart, followed by sacrifice either 10 or 30 days after BrdU injection. We then examined cells for coincident labeling of both BrdU and olfactory marker protein (OMP), a marker of neuronal maturity (Figure 4C). Although the absolute number of BrdU-labeled cells declines with age, we observed that the percentage of cells incorporating BrdU that reach maturity (OMP-positive) were consistent across all adult age groups 10 days (Figure 4A) or 30 days (Figure 4B) after BrdU administration. However, we did observe that olfactory epithelia examined from 1 month old mice exhibited a lower percentage of mature OSNs co-expressing BrdU. This is likely due to the prolonged bout of post-natal growth (Murdoch and Roskams, 2007), and the expected overproduction of neurons that are pruned during activity-dependent development. We suggest that 1 month old animals should not therefore be considered “adult” as is fairly common in the literature, at least for the purposes of regeneration studies. Hence, we find that cells born at advanced ages have the same potential to mature as those born at young adult ages.

### Lesion induces expansion and a morphological switch of the HBC population even at advanced ages

The identity of the neural stem cell in the adult olfactory epithelium is somewhat controversial as both types of basal cells are neurogenic progenitor cells (Graziadei and Graziadei, 1979). However, recent evidence indicates that the HBC is capable of regenerating all cell types in the epithelium following a severe lesion (Leung et al., 2007; Iwai et al., 2008). Like most stem cells, the HBC is relatively quiescent. During normal neuronal turnover, the GBC is the cell type that is normally identified by BrdU uptake (see Figure 1). In the adult OE, flattened HBCs are adherent to the basal lamina and form a single layer (Packard et al., 2011). However, it is unclear whether the HBCs in aged mice are genetically different from those that participate in early post-natal proliferation, as aging may disrupt the expression of regulators of the cell cycle (Legrier et al., 2001).

**Figure 5:**
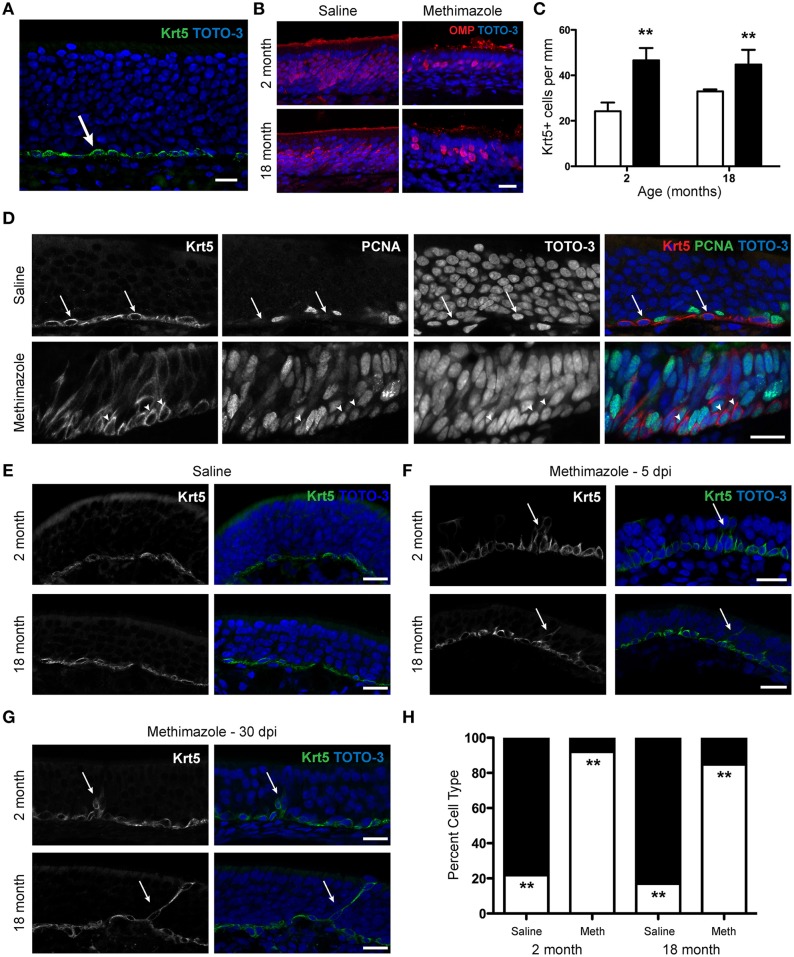
**Methimazole lesion of the olfactory epithelium causes an alteration in the morphology of horizontal basal cells in young and aged mice. (A)** Expression of Krt5, a member of the cytokeratin family, is restricted to the base of the olfactory epithelium and is a marker of the horizontal basal cells (green). Olfactory epithelium from a 1 month old male mouse is shown, with TOTO-3 (blue) nuclear stain also shown in the merged image. **(B)** Administration of Methimazole (50 mg/kg) causes an acute lesion of the olfactory epithelium. In young (2 month old) and aged (18 month old) mice the degree of sensory neuron loss was similar in olfactory epithelia examined 5 days post-injection (right) when compared to age-matched saline-injected control mice (left). **(C)** Following lesion with methimazole, the number of HBCs increases significantly 5 days after lesion (black bars) in both young (2 month old) and aged (18 month old) mice when compared to age-matched saline-injected control mice (white bars). Images such as those shown in **(E–G)** were used for quantification. Saline-treated mice: 2 month, 24.2 ± 3.8 Krt5+ cells per mm, *N* = 3; 18 month, 33.0 ± 0.9 Krt5+ cells per mm, *N* = 3. Methimazole-treated mice 5 dpi: 2 month: 46.6 ± 5.4 cells per mm, *N* = 3; 18 month, 44.8 ± 6.5 cells per mm, *N* = 3. Two-Way ANOVA for effects of age (*p* > 0.05) and treatment (^**^p < 0.01). **(D)** The alteration of morphology of HBCs following methimazole lesion is associated with a switch from a quiescent to a proliferative state. (*Top*) A marker of proliferation, PCNA (Proliferating Cell Nuclear Antigen) does not typically overlap that of Krt5-expressing HBCs in saline-treated mice (*arrows*). (Bottom) PCNA expression is observed in Krt5+ cells 5 days after methimazole injection (*arrowheads*). **(E)** In young and aged saline-treated mice, the typical HBC morphology is a flattened cell type along the base of the olfactory epithelium. Flat cells, 2 month: 137.7 ± 17.9 μm^2^, *N* = 3; 18 month: 103.7 ± 8.4 μm^2^, *N* = 3. **(F)** In young and aged mice examined 5 days after methimazole injection (5 dpi), the morphology of the HBCs has altered to an elongated, pyramidal morphology. Pyramidal cells, 2 month: 165.0 ± 4.4 μm^2^, *N* = 3; 18 month: 198.3 ± 32.5 μm^2^, *N* = 3. Data were analyzed by Two-Way repeated measures ANOVA for the effects of age (*p* > 0.05) and morphology (*p* < 0.03). Often, cells are observed to extend processes toward the apical surface of the olfactory epithelium (arrows). **(G)**, The same as in **(F)** but 30 day post-methimazole injection (30 dpi). The frequency of the pyramidal morphology has declined, but in both young and aged mice the elongated, proliferative morphology is still observed (arrows). **(H)** Following lesion with methimazole, the shape of HBCs changes significantly 5 days after lesion in both young (2 month old) and aged (18 month old) mice when compared to age-matched saline-injected control mice. In saline-injected mice, regardless of age, a significant majority of HBCs were flat in morphology (2 month: 78.2 ± 10.7%, *N* = 3; 18 month: 83.1 ± 9.0%, *N* = 3; black bar). In methimazole-injected animals, a significant majority of HBCs were pyramidal in morphology (2 month: 92.0 ± 2.0%, *N* = 3; 18 month: 84.8 ± 9.1%, *N* = 3; white bar). Two-Way repeated measures ANOVA for the effects of age (*p* > 0.05) and treatment (^**^p < 0.002). Scale bar, 20 μm.

Using a specific marker of HBCs in the olfactory epithelium, cytokeratin 5 (Krt5), we found that, as expected, in intact epithelium the HBC population is found restricted to the base of the epithelium with a flattened morphology (Figure 5A). Krt5 has been used as an “unambiguous” marker of HBCs (Calof and Chikaraishi, 1989); see also (Bergman et al., 2002; Carter et al., 2004; Williams et al., 2004; Schnittke et al., 2015). In addition, GBCs are cytokeratin 5/14 negative (Suzuki and Takeda, 1993) and HBCs are the “only cells in the olfactory mucosa that express these specific cytokeratins” (Holbrook et al., 1995). Krt5-CrePR mice have been used for lineage tracing the olfactory epithelium (Iwai et al., 2008) and microarray analysis verified that “that transcripts known to be preferentially expressed by HBCs” such as Krt5 and Krt14 were enriched in the HBC population (Fletcher et al., 2011). Previous work has indicated that severe chemical lesions (such as Methyl Bromide or Methimazole) that deplete neuronal precursors and mobilize GBCs are necessary to induce HBC proliferation, while neuron-specific lesions such as OBX do not recruit HBCs but rather induce GBC proliferation and differentiation (Leung et al., 2007) although a second result supported the conclusion that HBCs proliferate during neuronal turnover in intact mice as well as in response to injury induced by OBX (Iwai et al., 2008). We chose to administer 50 mg/kg methimazole (1-methyl-2-mercaptoimidazole; intraperitoneal injection; Genter et al., 1995), an anti-thyroid drug commonly used to severely lesion the olfactory epithelium and induce neurogenesis, to assess whether the HBC population would expand in response to a lesion challenge in aged mice. We selected this chemical lesion paradigm in this experiment as it would extensively deplete mature sensory neurons and other cell types in the epithelium, cause a subsequent depletion of the GBC population, and would certainly mobilize an HBC-mediated regenerative response (Leung et al., 2007). The extent of the lesion was assessed with immunohistochemistry for OMP; in young and aged mice the degree of sensory neuron loss was similar in olfactory epithelia examined 5 days post-injection (5 dpi, Figure 5B). In saline-treated animals examined 5 dpi, we found no difference in the number of HBCs observed in young vs. aged mice. When we examined methimazole-treated animals 5 dpi, the number of HBCs had significantly increased (Figure 5C). This effect was observed in both young adult and aged mice, and variation was not due to the interaction between age and drug treatment.

In aged mice, as the olfactory epithelium recovered from the lesion, HBC number declined; by 30 days post-lesion, HBC number in methimazole-treated animals was no different from saline-injected control animals (data not shown). We did not observe a decline in HBC number by 30 days post-lesion in young animals, however; in 2 month old methimazole-treated animals, HBC number remained elevated above that observed in saline-treated animals (data not shown). Hence it is possible that while the olfactory epithelium is recovering in aged animals, the percentage of newborn cells surviving is higher than that in young animals, as previous data indicate the aged OE is in fact capable of a robust response to a prolonged surgical lesion (Figure 2). Regardless, the HBC is found at the same frequency in saline-treated aged animals as is observed in young animals, and, in the face of a severe lesion, is capable of self-renewal, a critical stem cell requirement (Weiner, 2008).

Interestingly, we also observed that immediately following methimazole lesion, the Krt5-immunoreactive population exhibits a profound morphological change. After methimazole treatment, the typical flattened morphology of HBCs is altered, with a proportion of cells extending processes toward the apical surface of the epithelium (Figure 5E). This resembles a radial glial phenotype observed in other neurogenic regions of the nervous system (Morrens et al., 2012; Malatesta and Götz, 2013) and has been noted by other investigators as well (Carter et al., 2004) but to our knowledge this has not yet been demonstrated in aged mice. We further examined this phenomenon by investigating whether the alteration in morphology indicated a switch from a quiescent phenotype to a proliferative (mitotic) state. We therefore categorized Krt5^+^ immunoreactive cells as having either a flat or pyramidal morphology. Cells exhibiting pyramidal morphology more frequently expressed a marker of proliferation, PCNA (proliferating cell nuclear antigen), than cells with a flattened morphology (Figure 5D). Hence, the morphological switch itself is indicative of proliferative state, and epithelia that exhibit primarily pyramidal HBCs are highly proliferative.

We next sought to quantify whether this morphological change occurred with the same frequency in both young and aged animals when challenged with a methimazole lesion. We again categorized HBCs as having either a flat or pyramidal morphology in saline- and methimazole-injected young (2 month) and aged adult (18 month) mice. When we analyzed cross-sectional area according to morphology, we found a significant increase in cell area from a flat to a pyramidal morphology (data not shown). We also observed that in saline-injected mice, regardless of age, a significant majority of HBCs were flat in morphology while in methimazole-injected animals, a significant majority of HBCs were pyramidal in morphology (Figures 5E,F,H). We continued to observe an elongated morphology of a small portion of HBCs 30 days after lesion in both young and aged mice (Figure 5G). Thus, the morphological switch of the HBC from a flattened to pyramidal cell type is associated with a proliferative state and is observed even at very advanced ages.

## Discussion

Neural regeneration is rare in the nervous system, but could have an important impact on recovery from traumatic or pathologically induced brain injuries and on degenerative processes of normal aging. The potential of the basal stem cells to generate sensory neurons in a young adult olfactory epithelium (OE) has been known for more than 40 years, as they continually send new fledgling axons to stereotyped positions in the OB with each cell birth and growth cycle (Graziadei, 1973; Graziadei and Monti Graziadei, 1983). Until recently, virtually all studies concerning the regeneration of olfactory sensory neurons were on young adult animals. Here, we further the investigation of the dynamics of neurogenesis in the aged olfactory system. We find that neural stem cells in the aged olfactory epithelium do in fact possess a robust long-lasting proliferative capacity and can give rise to mature neurons.

We endeavored to answer several questions. First, does proliferation in the olfactory epithelium continue at a meaningful rate throughout life? Our studies in the VNO indicated that growth-associated proliferation declined with age, but that neuronal replacement continued well into advanced ages. In our previous study, we were able to separate the two processes based upon the unique architecture of the VNO. Here, in the olfactory epithelium, we demonstrate that normal cell proliferation also declines with age. However, we are unable to separate the processes of growth and cell replacement as clearly in this tissue due to the complex three-dimensional structure of the olfactory epithelium. We would note that proliferation near points where the olfactory epithelium ended and respiratory epithelium began, or in the turbinates of zones II-IV, was elevated in young animals in particular. This indicates the decrease in proliferation observed between 2 and 6 months of age is in fact evidence that growth-associated proliferation specifically declined with age, consistent with previous studies (see Murdoch and Roskams, 2007). However, regardless of total numbers of cells generated, neurogenesis was maintained at a constant rate. In support of this conclusion, when we queried whether the daughters of neural stem cells in aged animals could mature properly, we found that the newborn neurons co-expressed a marker of maturity (OMP) at the same rate in both young and aged animals. These data indicate that the fate and choice decisions faced by newborn neurons in aged animals are similar to those made by their counterparts in young animal.

The regenerative capacity of the olfactory epithelium following lesion has been studied quite extensively with several paradigms, including target ablation, sensory deprivation (unilateral naris occlusion), axotomy, and chemical ablation of the OE. Our next question was whether the aged OE could robustly respond to injury paradigms. We chose to use two separate lesion techniques, namely a surgical (OBX) and a chemical (Methimazole) ablation.

When we lesion the olfactory epithelium acutely by OBX, the basal cells return to action and initiate a significant increase in the basal rate of proliferation. Thus, although the basal stem cells in aged animals have become quiescent, they retain the capacity to meet the challenge of traumatic cell loss—even in 24 month old mice. When challenged with a sustained OBX injury, increased proliferation persists in aged mice, as in younger animals. These data demonstrate that certainly the proliferative capacity and perhaps the regenerative capacity of the epithelium remains intact with aging and that there is a viable population of neural stem cells even in very old animals. Suzukawa et al. (2011) suggest that recovery of the OE is incomplete with a methimazole-based lesion in aged animals. We find that with a lesion of mature neurons only by OBX, and therefore with limited epithelial damage beyond the mature neuronal population, the rate of neurogenesis in young and aged mice is comparable. However, while the ratio of proliferation in OBX vs. control animals is increased in all age groups, the total amount of proliferation in aged mice is in fact lower. Future experiments will need to explore this further, such as those experiments exploring the time required to regenerate the entire olfactory epithelium after injury at different ages. As OBX necessitates the removal of the olfactory bulb, such studies cannot be examined in this lesion model, as the rate neuron turnover remains high without a synaptic target for newborn sensory neurons.

We are particularly interested in the behavior of the HBC population of stem cells. While these cells are particularly active during the first third of the life of the animal, apparently for the purpose of continued post-natal growth of the tissue, they enter a quiescent phase at early post-natal stages, where previously pyramidal shaped basal cells change to a flattened morphology and complete lack of incorporation of the proliferative marker BrdU (Suzuki and Takeda, 1991b). The basal cells appear virtually inert in this condition. However, given the proper stimulation—that is death of olfactory sensory neurons due to injury or pathology—they transform both morphologically and functionally into robust stem cells. While this has been documented in young animals (Hurtt et al., 1988; Suzuki and Takeda, 1991a), we demonstrate for the first time this profound morphological change in aged animals undergoing an injury response. It is tempting to imagine that the basal stem cells are being stored in this “suspended” condition against the possible need for recovery in later life.

The results presented here, in concert with data from other laboratories, present numerous interesting questions. First, what is the biochemical switch that regulates the proliferative vs. quiescent state of HBCs? Recently it has been demonstrated that p63 down-regulation acts as a switch to activate HBCs following injury with the olfactotoxic gas methyl bromide (Schnittke et al., 2015), but it is not known what maintains the HBCs as a stem cell population. Second, are cells generated in older animals functionally comparable to those in young animals? Third, are newborn neurons in aged animals able to form appropriate synapses and new circuits? It is interesting to note that the function of mitral cells in the OB affect the number of sensory neurons surviving (Weiler and Farbman, 1999; Cavallin et al., 2010). A natural question is whether OB dysfunction observed in aging and neurodegenerative disease affects peripheral sensory neuron lifespan and regeneration (Curcio et al., 1985; Struble and Clark, 1992; Kovács, 2004). Finally, how are growth and regeneration linked—or separated? If the aged stem cell retains the capacity to make new neurons at advanced ages, what signal(s) cause the slowing of growth of the olfactory epithelium?

We have provided here a perspective of neuronal regeneration in the olfactory epithelium that is different from that seen anywhere else in the nervous system. Particularly the dual profile of the HBC documented here may lead to new and intriguing mechanisms for maintaining stem cell growth potential in aging neuronal tissue.

### Conflict of interest statement

The authors declare that the research was conducted in the absence of any commercial or financial relationships that could be construed as a potential conflict of interest.
